# Risk factors for relapse in patients with first-episode schizophrenia: Analysis of the Health Insurance Review and Assessment Service data from 2011 to 2015

**DOI:** 10.1186/s13033-018-0187-1

**Published:** 2018-03-02

**Authors:** Sang-Uk Lee, Minah Soh, Vin Ryu, Chul-Eung Kim, Subin Park, Sungwon Roh, In-Hwan Oh, Hye-Young Lee, SungKu Choi

**Affiliations:** 1 Department of Mental Health Research, National Center for Mental Health, Seoul, South Korea; 2 Department of Psychiatry, Hanyang University College of Medicine, Seoul, South Korea; 3 Department of Preventive Medicine, School of Medicine, Kyung Hee University, Seoul, South Korea

**Keywords:** First-episode schizophrenia, Relapse, Medical institutions

## Abstract

**Background:**

Schizophrenia is a recurrent, debilitating disease that is rarely curable. Rapid intervention after the first episode of schizophrenia has been shown to positively affect the prognosis. Unfortunately, basic data is scarce on first-episode schizophrenia in Korean patients making it difficult to create a comprehensive list of risk factors for relapse. This study aims to investigate the demographic characteristics and institutional factors of patients with first-episode schizophrenia in order to identify risk factors for relapse.

**Methods:**

Data from the Health Insurance Review & Assessment Service (HIRA) was used for this study to represent the Korean patient population. To identify factors affecting relapse, we explored gender, age, geographic location, medical benefits, type of medical institution, type of medication used, medication adherence, and the severity of symptoms. Data analysis was performed using the Cox proportional hazard model.

**Results:**

The number of patients diagnosed with first-episode schizophrenia in Korea over a 2-year period was 4567 of which 1265 (27.7%) patients experienced a relapse during the observational period. Factors affecting relapse included age, type of medical institution, type of medication used, medication adherence, and type of treatment (inpatient or outpatient) after the initial diagnosis, which varied depending upon the severity of symptoms.

**Conclusions:**

It was found that environmental and institutional factors as well as the type of medical treatment were crucial in determining whether patients with first-episode schizophrenia subsequently relapsed. The results of this study can be utilized as source material for directing therapeutic interventions and improving mental health policies in the future.

## Background

Schizophrenia has a prevalence of approximately 1%, and a considerable proportion of patients are resistant to treatment after they have progressed to the chronic stage of the disease. The quality of life of patients with chronic schizophrenia is diminished due to cognitive dysfunction, paresthesia, disordered thoughts, deterioration of emotional control, and negative symptoms [1, 2]. There is a great deal of evidence that early intervention should be conducted after the first episode of schizophrenia to ameliorate symptoms and improve the overall prognosis. The first 5 years is the most important period following the initial episode of a mental illness and is referred to as ‘the critical period’. Psychosocial and biological interventions performed during this period have been shown to positively influence the prognosis of the disease [3, 4]. Pharmacotherapy is considered to be the most important element of schizophrenia treatment, and roughly 80% of patients with first-episode schizophrenia improve within 6 months of starting medication [5, 6]. Gaebel et al. revealed that 91.5% of patients showed symptomatic remission, and 58.6% of patients remained in remission through their 1-year follow-up [7].

Once schizophrenia recurs, the psychiatric symptoms appear repeatedly, and hospitalization is recommended in many cases. The readmission rate is directly proportional to the rate of relapse, and hospitalization after treatment can thus be considered an index of relapse [8, 9]. One study reported that 41.0% of patients with schizophrenia were readmitted to a hospital within 1 year of discharge [10]. Another study indicated that 42.0% of patients with first-episode schizophrenia were readmitted to a hospital within 2 years [11]. Similarly, a Korean study also showed that 33.3–35.6% of patients with schizophrenia were re-hospitalized within 2 years [12, 13]. Collectively, the results from Lin et al., Sara et al., Kim et al. and Lee et al. studies may point to the possibility of relapse in patients with schizophrenia. Furthermore, relapse worsens a patient’s future response to treatment, lowers their quality of life, leads to social isolation, and raises social costs [14]. Relapse in younger patients with schizophrenia can have an even greater impact on their future lives, separating them from schools, workplaces, friends, and social communities [15]. Treatment with an antipsychotic drug is the most promising strategy for relapse prevention, and the risk of relapse is inversely proportional to medication adherence. Therefore, many studies have explored the correlation between relapse and treatment-related variables such as medication adherence [16–18]. There are, however, no studies examining institutional factors such as the type of medical institution where treatment was provided. Moreover, studies on relapse are limited to a one hospital or outpatients based study in Korea. This study investigated demographic characteristics and institutional factors of patients with first-episode schizophrenia in order to identify risk factors for relapse. Data from the HIRA was used for this study to better represent the Korean patient population.

## Methods

### Data

This is a retrospective study using health insurance claims data provided by the HIRA from January 2011 to December 2015. In Korea, 98% of the population is covered by the national health insurance, thus the claims data represent the whole population treated for a specific illness [19]. Based upon the health insurance claims data, patients included in this study were diagnosed with code F20 of International Classification of Diseases, 10th Revision (ICD-10). Patients who were not diagnosed with F20-29 in 2011–2013 but were then diagnosed with F20 after 2014 were defined as having first-episode schizophrenia for the purpose of this study [20]. The medications prescribed for each patient were found in the detailed drug statement. The medications were classified as either antipsychotic drugs or other drugs (antiepileptic drug, hypnotics & sedative drug). Antipsychotic drugs were then subdivided into monotherapy or combination therapy (2 or more drugs). Based upon methods used in previous studies, patients were grouped by medication adherence as follows: 0–50%, 50–80%, and more than 80% [21–23]. Medical institutions providing the treatment were classified into three groups according the number of beds: clinics have fewer than 30 beds, hospitals have 30–99 beds, and tertiary hospitals contain more than 99 beds. In the Korean mental health care system, tertiary hospital usually treats the patients with acute schizophrenia. But overall links between parts of the health system are not strong and there is little referral pathway. Therefore, patients usually choose the medical institutions that they think most appropriate [24]. Thus, there is little difference among medical institutions regarding type of patients, diagnosis and etc. To estimate the severity of symptoms, any patient who received outpatient treatment without hospitalization following their initial diagnosis of schizophrenia was considered to be a mild case.

### Medication adherence

Antipsychotic drug adherence was measured using a method proposed by Steiner and Prochazka in 1997 [23]. In this method, (A) is defined as the total number of days from initial diagnosis until either hospitalization or observed termination of the first medication, and (B) is defined as the number of days treated with a medication. The ratio of B to A is then referred to as the ‘adherence’. Patients were then divided into one of three groups based on their calculated adherence: 0–50%, 50–80%, or more than 80%.

### Selecting subjects

**Fig. 1:**
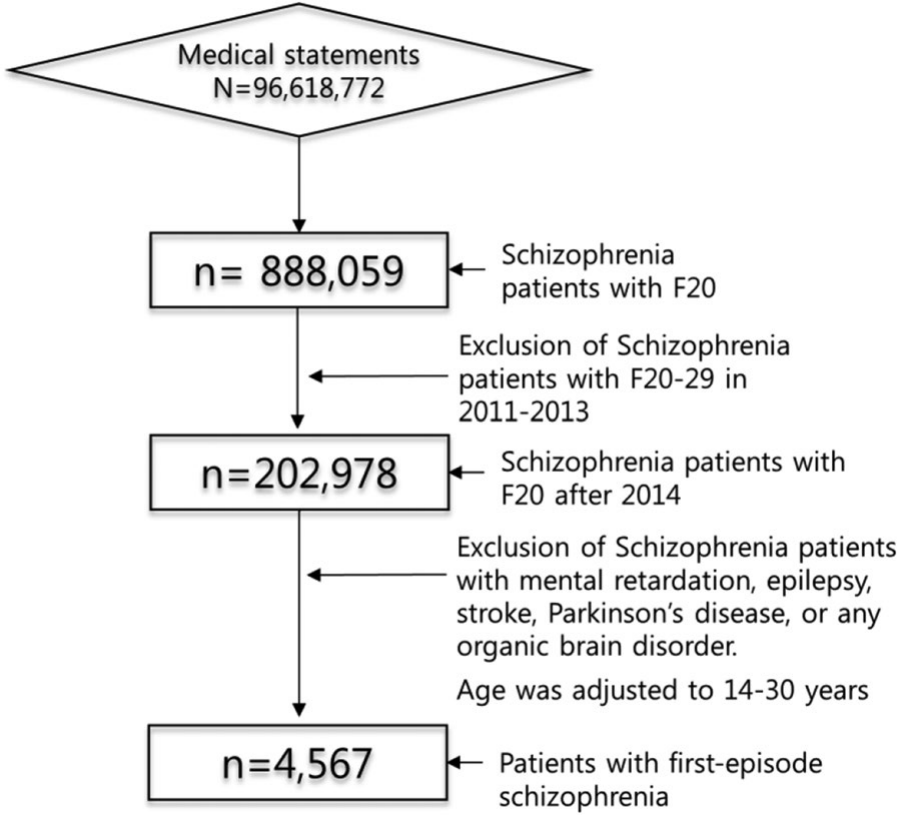
Flow diagram of the selection process for patients with first-episode schizophrenia

A total of 96,618,772 claims with a diagnostic code of F20-29 from the HIRA from January 2011 to December 2015 were analyzed. Of these, 202,978 patients who were not diagnosed with F20-29 from 2011–2013 but were diagnosed with F20 in 2014–2015 were selected. Using standards established by prior studies, only patients aged 14–30 years old at the time of their initial diagnosis were included [25]. In order to increase the diagnostic accuracy, patients diagnosed with F20 more than twice were included in this study only if they were outpatients. Patients who also carried a diagnosis of mental retardation, epilepsy, stroke, Parkinson’s disease, or any organic brain disorder were excluded. This yielded a final study population of 4567 patients (Fig. 1).

### Statistical analysis

The observation period for relapse in the 4567 patients selected with first-episode schizophrenia took place between January 2014 and December 2015. The observation period was scored on a monthly basis. We defined admission as an event, in the survival analysis. Admission was regarded as a result of relapsed schizophrenia [9]. Patients who relapsed were scored ‘1’ and those who did not relapse were scored ‘0’ for survival analysis. To identify the factors affecting relapse, the following variables were used: gender, age, geographic location, medical benefits, type of medical institution, type of medication used, medication adherence, and type of treatment (inpatient or outpatient) after the initial diagnosis.

The risk factors affecting relapse were analyzed by the Cox proportional hazard model. This technique does not assume any particular distribution, because it is impossible to presume a theoretical distribution of subjects’ survival time. As such, the Cox model is advantageous in that the data’s baseline hazard function provides a stable estimate of coefficients [26]. The hazard ratio (HR) and 95% confidential interval (CI) were measured through this COX proportional hazard model, and statistical significance was defined by a 5% confidential level using the two-sided test. SAS Enterprise Guide software version 6.1 (SAS Institute Inc., Cary, NC, USA) was used for all statistical analysis.

## Results

The average age of the 4567 subjects with first-episode schizophrenia was 23.12 years. Of these, 1265 (27.7%) of patients experienced a relapse during the observation period, and significant differences were found between groups according to the type of medical institution, type of medication used, medication adherence, and the severity of symptoms. Patient characteristics including gender, age, geographic location, medical benefits, type of medical institution, type of medication used, medication adherence, and the severity of symptoms are presented in Table 1.

**Table 1:** Demographical characteristics of patients with first-episode schizophrenia

Characteristics	Categories	No relapse		Relapse		(*P* value)
		n = 3302	72.30%	n = 1265	27.70%	
Gender	Male	1654	72.3%	635	27.7%	0.9485
	Female	1648	72.3%	630	27.7%	
Age (years)		3302	23.5 ± 4.4	1265	23.1 ± 4.3	0.038*
Geographic location	Metropolitan City	1727	73.2%	632	26.8%	0.1461
	Province	1575	71.3%	633	28.7%	
Medical benefits	Non-recipient	3057	72.0%	1187	28.0%	0.1391
	Recipient	245	75.9%	78	24.1%	
Type of medical institution	Tertiary hospital	1135	70.8%	467	29.2%	< 0.0001**
	Community hospital	846	65.8%	440	34.2%	
	Clinic	1321	78.7%	358	21.3%	
Antipsychotic drugs	Monotherapy	1585	78.0%	448	22.0%	< 0.0001**
	Combination therapy	1442	70.5%	602	29.5%	
	Other drugs	275	56.1%	215	43.9%	
Medication adherence	0–50%	1176	64.1%	660	35.9%	< 0.0001**
	50–80%	452	78.7%	122	21.3%	
	More than 80%	1674	77.6%	483	22.4%	
Treatment after initial diagnosis	Outpatient treatment	2352	75.6%	759	24.4%	< 0.0001**
	Inpatient treatment	950	65.2%	506	34.8%	

* *P* < 0.05, ** *P* < 0.001

The average follow-up period was 11.8 months with a median of 12 months. The shortest and longest follow-up periods were 1 and 24 months, respectively. During the follow-up period of 4475.1 person-years, a total of 1265 subjects were admitted to hospitals, and the calculated rate of relapse by person-years is shown in Table 2.

**Table 2:** The rate of relapse (per 1000 person-years) by patient characteristics

Categories	N (%)	Relapse	Person-year	Relapse rate (per 1000 person-years)	95% confidence interval
Male	2289 (50.1)	635	2248.1	282.5	261.1–305.1
Female	2278 (49.9)	630	2227.0	282.9	261.4–305.6
Age	4567 (100.0)	1265	4475.1	282.7	261.3–305.5
Metropolitan city	2359 (51.7)	632	2332.9	270.9	250.4–292.7
Province	2208 (48.3)	633	2142.2	295.5	273.1–319.2
Non-recipient	4244 (92.9)	1187	4163.2	285.1	269.2–301.7
Recipient	323 (7.1)	78	311.9	250.1	199.0–310.4
Tertiary hospital	1602 (35.1)	467	1643.8	284.1	250.3–323.6
Community hospital	1286 (28.2)	440	1173.1	375.1	341.2–411.4
Clinic	1679 (36.8)	358	1658.2	215.9	194.4–239.1
Monotherapy	2033 (44.5)	448	2047.5	218.8	199.2–239.8
Combination therapy	2044 (44.8)	602	1928.8	312.1	287.9–337.8
Other drugs	490 (10.7)	215	498.8	431.0	376.2–491.6
0–50%	1836 (40.2)	660	1938.3	340.5	315.3–367.2
50–80%	574 (12.6)	122	583.0	209.3	174.5–249.0
More than 80%	2157 (47.2)	483	1953.8	247.2	225.9–270.0
Outpatient treatment	3111 (68.1)	759	3049.1	248.9	231.7–267.1
Inpatient treatment	1456 (31.9)	506	1426.0	354.8	324.9–386.8

### Risk factors for relapse in patients with first-episode schizophrenia

To analyze the variables affecting relapse in patients with first-episode schizophrenia, a Cox-regression was performed. After adjusting for the variables of gender, age, geographic location, medical benefits, type of medical institution, type of medication used, medication adherence, and the severity of symptoms, significant differences were found in the hazard ratio (HR) according to age, type of medical institution, type of medication used, medication adherence, and the severity of symptoms. The risk for relapse appeared low (HR of 0.96) in patients with a delayed age of onset. The HR of community hospitals compared with tertiary hospitals was 1.17, which was significantly high. The HR of clinic-level facilities compared with tertiary hospitals declined significantly to 0.75. The HR of other drugs compared with monotherapy with antipsychotic drugs was 2.09, which was significantly high. After dividing patients into three groups based on medication adherence, the HR of the group with 0–50% adherence compared with the group with more than 80% adherence was 1.59, a significantly high result. Using the severity of symptoms, the HR of the patient group who received inpatient treatment compared with the group who only received outpatient treatment after the initial diagnosis was 1.46, another significantly high result (Table 3).

**Table 3:** The Hazard Ratio for relapse by characteristics of patients with first-episode schizophrenia

Characteristics	Categories	Status		Univariate		Multivariate	
		No relapse	Relapse	Hazard ratio	95% CI	Hazard ratio	95% CI
Gender	Male	1654	635	1.00	(0.90–1.12)	0.95	(0.85–1.06)
	Female	1648	630	1.00 (reference)			
Age (years)		3302	1265	0.98*	(0.97–0.99)	0.96*	(0.95–0.98)
Geographic location	Metropolitan City	1727	632	1.00 (reference)			
	Province	1575	633	1.09	(0.98–1.22)	1.02	(0.92–1.14)
Medical benefits	Non-recipient	3057	1187	1.00 (reference)			
	Recipient	245	78	0.85	(0.68–1.07)	0.85	(0.68–1.07)
Type of medical institution	Tertiary hospital	543	230	1.00 (reference)			
	Community hospital	846	440	1.26*	(1.10–1.43)	1.17*	(1.02–1.33)
	Clinic	1321	358	0.73*	(0.63–0.83)	0.75*	(0.63–0.90)
Antipsychotic drugs	Monotherapy	1585	448	1.00 (reference)			
	Combination therapy	1442	602	1.40*	(1.24–1.58)	1.04	(0.88–1.21)
	Other drugs	275	215	2.29*	(1.95–2.70)	2.09*	(1.77–2.46)
Medication adherence	0–50%	1176	660	1.60*	(1.42–1.80)	1.59*	(1.41–1.80)
	50–80%	452	122	0.88	(0.72–1.07)	0.94	(0.77–1.15)
	More than 80%	1674	483	1.00 (reference)			
Treatment after initial diagnosis	Outpatient treatment	2352	759	1.00 (reference)			
	Inpatient treatment	950	506	1.50*	(1.34–1.67)	1.46*	(1.24–1.71)

Multivariate hazard ratio was calculated including all variables in the table

*CI* confidence interval

* *P* < 0.05

### Risk factors for relapse in patients with first-episode schizophrenia by severity

**Fig. 2:**
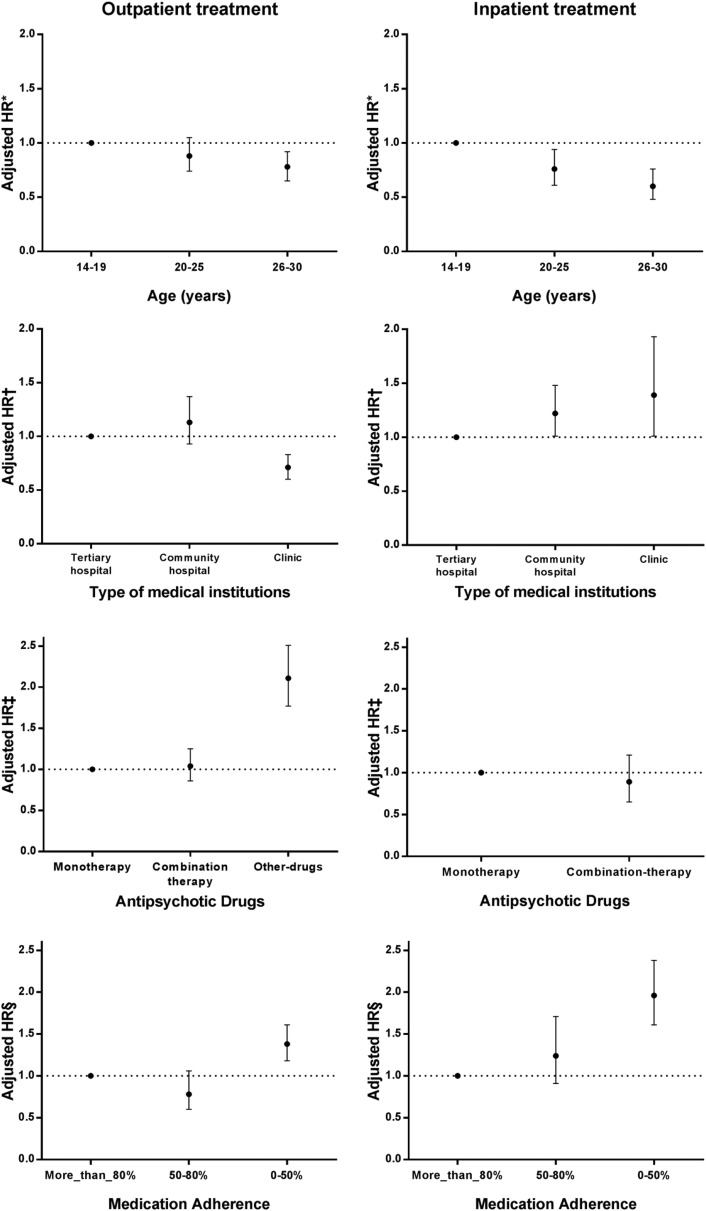
The Hazard Ratio for relapse by characteristics of patients with first-episode schizophrenia, stratified by severity. *HR* Hazard Ratio. *Adjusted for type of medical institution, type of medication used, and medication adherence. ^†^Adjusted for age, type of medication used, and medication adherence. ^‡^Adjusted for age, type of medical institution, and medication adherence. ^§^Adjusted for age, type of medical institution, and type of medication used

To estimate the severity of symptoms, we explored whether subjects received inpatient or outpatient treatment after the initial diagnosis. Patients were divided into two groups based on the type of treatment, and variables from each group were analyzed after being adjusted. Looking at age, the risk for relapse appeared low in patients with a delayed age of onset, regardless of the severity. In the outpatient treatment group, the HR of 26–30 year-olds compared with 14–19 year-olds was 0.78. In the hospital treatment group, the HR of 20–25 year-olds compared with 14–19 year-olds was 0.76, and the HR of 26–30 year-olds compared with 14–19 year-olds was 0.60. This indicates that the HR of patients aged 14–20 with first-episode schizophrenia was significantly high. With all other variables, the HR trended according to the severity of symptoms (Fig. 2).

In the patient group who received outpatient treatment in clinic-level facilities after the initial diagnosis, the relapse HR was 0.71 compared with those treated in tertiary hospitals, a significantly low result. The HR of patients treated with other mediations but not antipsychotic drugs compared with those treated with antipsychotic drugs was significantly high at 2.11. The HR of the group with 0–50% medication adherence compared with the group with over 80% adherence was 1.38, which was also significantly high.

The results of the patient group who received inpatient treatment after their initial diagnosis were somewhat different from the group who received only outpatient treatment. In the patient group treated in community hospitals and clinics, the relapse HR was 1.22 and 1.39, respectively, both of which were significantly high. No significant difference in the HR between monotherapy and combination therapy (2 or more antipsychotics) was found. The HR of the group with 0–50% medication adherence compared with the group with over 80% adherence was significantly high at 1.96.

## Discussion

This study was designed to identify factors affecting relapse in patients with first-episode schizophrenia. The total number of subjects included in this study was 4567 with a total follow-up period of 4475.1 person-years. During this observation period, 1265 patients relapsed. As a result, the factors showing a statistically significant effect on relapse were age, type of medical institution, type of medication used, medication adherence, and the severity of symptoms at the time of their initial diagnosis. In general, the HR of relapse tended to vary according to the severity of symptoms.

Looking at age, the risk for relapse appeared low in patients with a delayed age of onset. Although a previous study showed that there was no correlation between the age of onset and the risk of relapse, the present study revealed that a younger age of onset was associated with a worse prognosis [18, 27]. This observation, that the risk for relapse declined significantly with a delayed age of onset, suggests that symptoms are more severe and recurrence is more frequent in patients diagnosed at a young age. Similar results were observed even when the cases were divided by severity into inpatient and outpatient treatment groups. Wood et al. also reported the same outcomes [28]. To prevent relapse in patients with first-episode schizophrenia, early screening systems would therefore be indicated.

In the analysis by type of medical institution, the relapse HR of patients treated in community hospitals compared with those treated in tertiary hospitals was 1.17, a significantly high result. The HR decreased to 0.75 in those treated in clinics. These results may involve a selection error, however, as the type of medical institution attended after the initial episode of schizophrenia depended upon the severity of symptoms. To eliminate this possibility, we examined the data after separating patients into inpatient and outpatient treatment groups. In the outpatient group, the HR of treatment in clinics compared with treatment in tertiary hospitals yielded a significantly low result, indicating that mild cases typically visit clinics. In the inpatient group, the HR of treatment in community hospitals compared with treatment in tertiary hospitals was significantly high; inpatient treatment in clinics also showed a significantly high HR. These results may suggest that treatment in community hospitals and clinics is inferior to that provided by tertiary hospitals in terms of institutional and treatment conditions. In Korea, community hospitals and clinics are regarded as primary medical institutions, thus they are very similar from a functional point of view [29]. Lee et al. reported that the quality of Korean primary medical institutions (0.3) was much poorer than that of the UK (1.9) or Canada (1.2) [30]. In addition, the results reflect that tertiary hospitals are equipped better, because they can provide a variety of medical services compared with community hospitals and clinics in Korean healthcare system [31]. To prevent relapse in patients with first-episode schizophrenia, environmental and institutional reorganization will be required. In the Korean medical system, the private medical institutions play a major role, up to 94% [32]. In the health insurance system, the daily medical cost of treatment is fixed for Medicaid patients with schizophrenia. When the price is converted to cost per day, the costs for inpatient therapy are 50,000 won (about $44.1) [33]. In this system, the hospitals and clinics use low-cost drugs in order to increase profits. Therefore, the government needs to change the health insurance system to a fee-for-service policy. However, changing to a fee-for-service (FFS) system would require fundamental rebuild of the health insurance schemes. Thus, the government will need to consider an incentive system to improve quality of care such as quality assessment system. And it is necessary to conduct further studies to suggest more viable policy implications for the reform of the schizophrenia care system. However, including patients with health insurance, the conventional drug use compared with atypical drug use in clinics and hospital was higher than in the tertiary hospital setting [34]. This result suggests that psychiatrists operating private clinic may prefer to conventional medications, which may have an impact on treatment of patients with schizophrenia. Therefore, to improve the current uneven situation, continuing education system for psychiatrist should be strengthened as well.

The relapse HR for patients treated with other drugs compared with monotherapy with an antipsychotic was significantly high at 1.81, suggesting that antipsychotic drugs are more effective [35]. To prevent relapse, antipsychotic medications should be administered from the onset of schizophrenia, regardless of the severity of symptoms. Combination therapy with more than one antipsychotic medication is becoming more common in the treatment of schizophrenia, and its efficacy is excellent [36, 37]. There was, however, no difference in the relapse HR between monotherapy and combination therapy in this study. The result was the same after adjusting for the severity of symptoms. Thus, to prevent relapse, antipsychotic medications should be administered from the onset; providers must then decide whether to use monotherapy or combination therapy.

Analyzing relapse HR according to medication adherence, the HR of the low-level adherence group (0–50%) compared with the high-level adherence group (more than 80%) was 1.59, a significantly high result. This result was the same in both the inpatient and outpatient treatment groups. These results coincide with previous studies in that the risk of relapse decreases with a higher medication adherence [38, 39].

There are several limitations in this study. First, the data did not provide information about the actual severity of patient with schizophrenia. Therefore, this study performed subgroup analysis by classifying hospital treatment and outpatient treatment in order to exclude the effect of severity. However, it could not confirm effect of severity. Second, in this study, we used indirect measures of medication adherence. Thus, we were unable to confirm whether the patients complied with the prescribed medication or not. Further research is needed to investigate these limitations.

Despite these limitations, our study has a number of strengths. Using the HIRA data, this study confirmed the risk factor for relapse in all patients with first episode schizophrenia in Korea. The results of this study showed that necessity for change of structural, institutional factors as well as medical treatments in managing patients.

## Conclusions

This study investigated the risk factors for relapse in patients with first-episode schizophrenia using data from the HIRA in order to best represent the Korean patient population. Approximately 28% of the 4567 patients included in this study relapsed over 4475.1 person-years. After adjusting for demographic features, the factors which increased the risk of relapse include a younger age of onset, treatment in clinics and community hospitals, use of medications other than antipsychotic drugs, reduced medication adherence, and high severity of symptoms at the initial diagnosis. The results varied, however, when we stratified by the severity of symptoms. Consequently, the results of this study suggest the need for changes in the structure of care, institutional factors, and medical treatments used to manage patients with first-episode schizophrenia in Korea. It is our hope that this study can be utilized as source material for directing therapeutic interventions and improving mental health policies in the future.
