# Correction to: Risk factors for relapse in patients with first-episode schizophrenia: analysis of the Health Insurance Review and Assessment Service data from 2011 to 2015

**DOI:** 10.1186/s13033-018-0193-3

**Published:** 2018-06-07

**Authors:** Sang‑Uk Lee, Minah Soh, Vin Ryu, Chul‑Eung Kim, Subin Park, Sungwon Roh, In‑Hwan Oh, Hye‑Young Lee, SungKu Choi

**Affiliations:** 1 Department of Mental Health Research, National Center for Mental Health, Seoul, South Korea; 2 Department of Psychiatry, Hanyang University College of Medicine, Seoul, South Korea; 3 Department of Preventive Medicine, School of Medicine, Kyung Hee University, Seoul, South Korea

## Correction to: Int J Ment Health Syst (2018) 12:9 10.1186/s13033-018-0187-1

Following publication of the original article [1], the authors wrote to request that the title of their manuscript be changed from “Analysis of the Health Insurance Review and Assessment Service data from 2011 to 2015” to “Risk factors for relapse in patients with first-episode schizophrenia: analysis of the Health Insurance Review and Assessment Service data from 2011 to 2015”. The authors had asked the typesetters to make this change at proof correction stage, but owing to a misunderstanding, this change was not carried out.

The original article has been updated.
